# Multidrug-Resistant *Staphylococcus aureus*, India, 2013–2015

**DOI:** 10.3201/eid2209.160044

**Published:** 2016-09

**Authors:** Mohit Kumar

**Affiliations:** Biotechnology and Bioinformatics, NIIT University, Neemrana, India

**Keywords:** Staphylococcus aureus, antimicrobial resistance, multidrug-resistant, methicillin, linezolid, tigecycline, vancomycin, MRSA, LRSA, VRSA, TRSA, bacteria, India, multidrug-resistant Staphylococcus aureus, staphylococci

**To the Editor:** Methicillin-resistant *Staphylococcus aureus* (MRSA) is a versatile pathogen capable of causing a wide variety of human diseases. Increased frequency of *S. aureus* infections imposes a high and increasing burden on healthcare resources. In many countries, MRSA infections in hospitals are common. Data from the National Nosocomial Infections Surveillance system suggest that, in the United States, incidence of nosocomial MRSA infections is steadily increasing and that these infections account for >60% of intensive care unit admissions (*1*,*2*). *S. aureus* has developed resistance to several antimicrobial drugs, including second- and third-line drugs. Only a few drugs, such as vancomycin (a glycopeptide), daptomycin (a lipopeptide), and linezolid (an oxazolidinone), have been approved for the treatment of serious infections caused by MRSA. Another drug, tigecycline (a glycylcycline), has shown good activity against MRSA strains in vitro (*3*). The epidemiology of MRSA is constantly changing, which results in variation in its drug-resistance patterns throughout regions and countries (*4*). Therefore, to support clinicians in preventing and treating infection, epidemiologic surveillance is essential. We report resistance patterns of *S. aureus* collected over 2 years (December 2013–November 2015) from blood samples of patients admitted to 1 hospital in Odisha, eastern India.

A total of 47 *S. aureus* isolates were collected; only 1 isolate per patient was included in the study. Susceptibility of the isolates was tested against antimicrobial agents according to the Clinical and Laboratory Standards Institute broth microdilution procedure and interpretation criteria (http://clsi.org/). MICs for the isolates were confirmed by using a Vitek 2 Compact automated system (bioMérieux, Marcy l’Étoile, France). *S. aureus* ATCC 25923 was used as a control strain. *S. aureus* identification was confirmed by using a Vitek 2 system, by hemolytic activity on blood agar, and by positive catalase activity test results. Clinical MRSA isolates were analyzed by using PCR with specific primers: *mecA* (*5*), *cfr* (*6*), and *VanA* (*7*).

 Among the 47 *S. aureus* isolates, 28 (60%) were resistant to oxacillin (MICs 4–64 mg/L) and cefoxitin (MICs 8–64 mg/L). All MRSA isolates were able to grow in selective medium containing either aztreonam (75 mg/L) or colistin (10 mg/L). Screening of MRSA isolates showed that 2 isolates were highly resistant to vancomycin (MIC >100 mg/L) (Figure). Further screening showed that both vancomycin-resistant isolates were also resistant to linezolid (MIC >100 mg/L) (Figure). PCR amplification of both isolates indicated presence of all 3 genetic determinants: *mecA* (methicillin resistance), *cfr* (linzolid resistance), and *VanA* (vancomycin resistance). Among the 3 isolates that showed resistance to tigecycline (MIC >50 mg/L), 1 isolate was susceptible to vanocmycin and linezolid (Figure). Unlike previously reported isolates, these 2 MRSA isolates showed resistant phenotypes to linezolid, tigecycline, and vancomycin.

**Figure:**
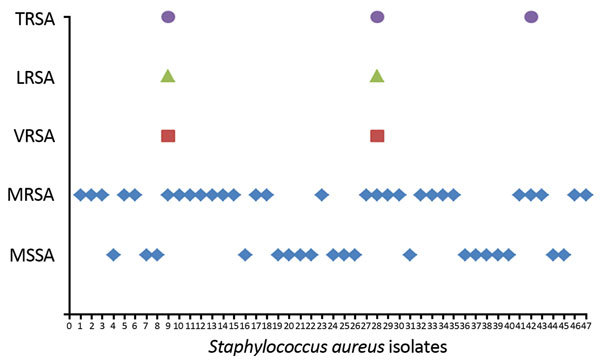
Distribution of various resistance types of *Staphylococcus aureus *isolates collected in eastern India, 2013–2015. LRSA, linezolid-resistant *S. aureus*; MRSA, methicillin-resistant *S. aureus*; MSSA, methicillin-sensitive *S .aureus*; TRSA, tigecycline-resistant *S. aureus*; VRSA, vancomycin-resistant *S. aureus*.

MICs observed in this study were higher than those previously reported. Vancomycin-resistant *S. aureus* has been identified in many other countries. Most linezolid-resistant *S. aureus* has been isolated from patients in North America and Europe (*8*). The tigecycline-resistant *S. aureus* isolate (MIC >0.5 mg/L) reported from Brazil was also susceptible to linezolid, teicoplanin, and vancomycin (*9*).

This study indicates the emergence of multidrug-resistant *S. aureus* with co-resistance to methicillin, vancomycin, linezolid, and tigecycline. Although the clinical significance of these findings is unknown, the decline in drug effectiveness against *S. aureus* infections represents a looming threat to patient health and highlights the possibility of a return to illness and death rates similar to those before antimicrobial drugs were available.
